# Bayesian Modeling of Polarizable Water: Lessons for Force Field Development

**DOI:** 10.1021/acs.jctc.6c00260

**Published:** 2026-05-23

**Authors:** Alfred T. Nordman, Stefan Engblom, David van der Spoel

**Affiliations:** † Department of Cell and Molecular Biology, 8097 Uppsala University, Uppsala 751 23, Sweden; ‡ Division of Scientific Computing, Department of Information Technology, Uppsala University, Uppsala 751 23, Sweden; § Science for Life Laboratory, Department of Information Technology, Uppsala University, Uppsala 751 23, Sweden

## Abstract

Computer simulation of molecular dynamics is useful only when the accuracy of models and the uncertainty of model predictions can be quantified. The accuracy of simulations can usually be established by comparing with reference data from experiments or quantum chemistry. However, trade-offs in designing simulation models often lead to property-dependent accuracy. For instance, models trained to reproduce liquid-phase density and enthalpy of vaporization do not automatically reproduce solid or gas-phase properties. This means that the method chosen for force field design introduces uncertainties in the final model, which are often ignored. Although the statistical uncertainty due to stochastic simulations is usually accounted for, the uncertainty due to parameters is harder to quantify. In a recent paper(npj Comput. Mater. 11 (2025), 366).10.1038/s41524-024-01501-5PMC1176107239872024, we developed Bayesian three-point water models based on synthetic likelihoods. The results were shown to depend strongly on the reference observables used. Here, we extend this work to polarizable models by applying Bayesian inference to the SWM4-NDP model (Chem. Phys. Lett. 418 (2006), 245). We consider two van der Waals functional forms, and subject their corresponding parameters to Markov-chain Monte Carlo sampling. The two functional forms have similar performance despite the differences in their complexity, but favor different inference observables. In addition to this, we find that explicit polarization does not significantly reduce parameter uncertainty for the chosen observable set. We then compare models identified by Bayesian inference with models in which the van der Waals potential was trained on gas-phase dimer energies from symmetry-adapted perturbation theory using the Alexandria Chemistry Toolkit (Digit. Discovery 4 (2025), 1925).Intriguingly, the Lennard-Jones 12–6 parameters trained on the gas-phase dimers are very similar to the optimal parameter set from the Bayesian inference, and the resulting models have similar accuracy. Replacing the Lennard-Jones 12–6 potential with the Wang-Buckingham potential (J. Chem. Theory Comput. 9 (2013), 452)26589047 10.1021/ct300826tand training on gas-phase data yields an unstable model, whereas the corresponding optimal Bayesian model gives reasonable results. A further analysis shows that observables respond in different ways to changes in force field parameters, highlighting the need to consider a large number of observables during force field training. Implications for model development as well as uncertainty quantification are discussed.

## Graphical abstract

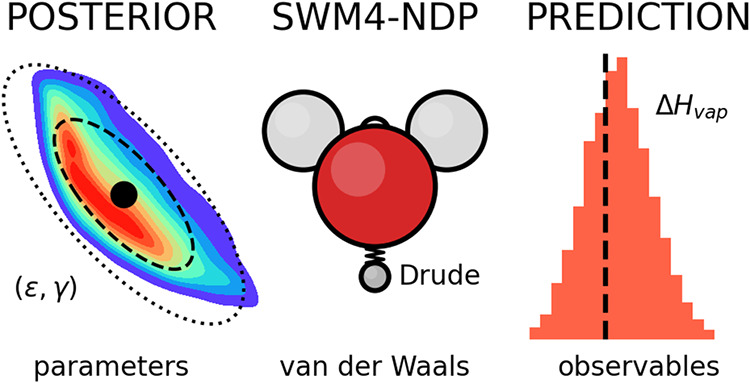

## 1 Introduction

Reliable molecular simulations require not only accurate force fields but also a principled treatment of uncertainty.^1–6^ Force field parameters are typically inferred from limited and noisy data, yet are almost universally deployed as fixed point estimates, implicitly assuming a level of certainty that is rarely justified in practice, particularly given the well-known limitations of fixed functional forms and the associated lack of parameter uniqueness in classical force fields.^7^ Bayesian inference offers a transparent alternative by treating parameters as probability distributions, enabling uncertainty to be quantified and propagated through predicted observables.^8,9^ Such perspectives have also been advocated in the broader force field community as a means to address parameter degeneracy and functional-form uncertainty in molecular simulations.^10^ This probabilistic perspective is particularly important in molecular simulations, where model responses are often highly sensitive to parameter values, such that even modest perturbations can lead to qualitatively different predicted behavior.

In molecular dynamics simulations, likelihood functions are often analytically intractable, making direct Bayesian inference challenging. Synthetic likelihood^11^ (SL) based methods address this limitation by constructing statistically motivated likelihood approximations from simulation-derived statistics. Provided that these statistics are approximately normally distributed, SL-based methods offer a rigorous and computationally feasible approximation that integrates naturally with Markov-chain Monte Carlo (MCMC) sampling, e.g., the Metropolis–Hastings^12^ (MH) algorithm. This approach avoids the need for explicit surrogate models^13^ or reweighting schemes,^14^ while remaining flexible enough to incorporate diverse and heterogeneous experimental data and still preserve a direct connection to the underlying molecular simulations.

The need for uncertainty-aware inference becomes especially apparent for water models, owing to the central role of water as a solvent and reference system across a wide range of molecular simulations and thermodynamic conditions. Widely used fixed-charge descriptions, such as the TIP3P, TIP4P^15^ and related models, remain attractive thanks to their computational efficiency, but their restricted functional form leads to systematic biases that are difficult to eliminate through parameter optimization alone,^16,17^ often requiring molecular geometries that deviate from gas-phase reference values to achieve improved agreement with condensed-phase properties.^14,18^ Polarizable water models extend this functional form by allowing molecules to respond to their local electrostatic environment, thereby implicitly capturing many-body effects through a physically motivated mechanism.^19^ Embedding such models within a Bayesian SL-based framework enables their increased expressiveness to be assessed quantitatively, not only in terms of improved agreement with experiment but also through a rigorous characterization of the remaining parameter uncertainty and functional-form limitations.

In this work, we extend our Bayesian SL-based framework from nonpolarizable, three-point water models to a polarizable description of liquid water. Building on our earlier study^20^ of TIP3P^15^-like models, where we quantified uncertainty and model limitations within a fixed-charge functional form, we now investigate how those conclusions change when explicit molecular polarizability is introduced. Polarizable descriptions of water have a long history, with early shell-model approaches explicitly incorporating electronic response to improve physical realism and phase transferability.^21,22^ This shift allows us to assess whether increased physical expressiveness translates into reduced model bias and improved consistency across observables, or instead introduces different forms of parameter correlation and reduced parameter interpretability.

Specifically, we consider the Simple Water Model with four sites and a negative Drude particle (SWM4-NDP), a polarizable water recipe introduced by Lamoureux et al.^23^ and subsequently adopted as a reference water model within Drude-based polarizable force fields.^24,25^ We focus on Bayesian inference of its van der Waals parameters, allowing us to study a well-documented polarizable water system while explicitly accounting for polarization. More recent polarizable variants, including OPC3-pol,^26^ SWM4-HLJ,^27^ and SWM3,^28^ have been proposed to address different physical and computational trade-offs within polarizable water models; however, SWM4-NDP remains widely used within Drude-based polarizable force fields and well characterized, making it a suitable and relevant test case for assessing uncertainty and parameter identifiability in polarizable force fields. Alongside the standard Lennard-Jones 12–6 functional form, we also examine an alternative in which the short-range repulsion is described by Wang’s modified Buckingham potential,^29^ referred to here as the Wang–Buckingham functional form. This choice is motivated by its demonstrated impact on phase transferability in polarizable force fields with complex short-range interactions.^30^ By comparing the resulting posterior distributions and predictive uncertainties, we assess how polarizability and choice of functional form affect the extent to which the chosen observables constrain model parameters, uncertainty propagation, and overall adequacy of the model within a fully Bayesian framework. The purpose of the present Bayesian framework is therefore not primarily to identify a single optimal force field, but rather to determine how strongly the chosen observables constrain the parameter space, and what this implies for parameter identifiability and trade-offs within a fixed functional form.

An unsolved problem in systematic force field design is whether molecular models derived from gas-phase data can be applied to the condensed phase.^10^ Although highly detailed water models exist that explicitly account for many-body interactions,^19^ there still is a need to have models of “intermediate” complexity, that is, more accurate than point-charge and a Lennard-Jones 12–6 potential,^31^ but simpler and more general than many body models.^32^ While it is not the purpose of this work to derive new water models to replace SWM4-NDP, it is nevertheless informative to compare the maximum a posteriori (MAP) parameter estimates with those obtained from training on gas-phase data, in particular dimer energies. In doing so, we hope to gain insight into whether parameters favored by gas-phase properties differ from those favored by liquid-phase simulations.

## 2 Methods

### 2.1 Water Model Description

In this work we adopt the SWM4-NDP model originally introduced by Lamoureux et al.^23^ The model is employed here not as a target for reparameterization, but as a well-established and physically motivated test case for examining Bayesian parameter inference and uncertainty quantification in polarizable force fields. The model represents each water molecule using a rigid three-atom geometry with two additional sites, namely a massless virtual site positioned along the H–O–H bisector at a fixed distance from the oxygen, and a Drude particle harmonically bound to the oxygen atom to represent electronic polarization.^33^ The permanent molecular dipole is constructed analogously to conventional four-site fixed-charge descriptions, while polarization emerges dynamically through the displacement of the Drude particle in response to the local electrostatic environment.

The polarizability is controlled by the Drude charge and the harmonic restoring force, such that the induced dipole responds linearly in the weak-field limit. All intramolecular geometrical parameters and electrostatic terms are kept fixed throughout this work, consistent with the original parametrization of the SWM4 family; this isolates the role of the short-range van der Waals interaction in determining posterior structure and predictive uncertainty. Nonbonded interactions between oxygen sites are described using the Lennard–Jones 12–6 ^31^ (LJ) potential with long-range dispersion corrections, while electrostatics between all sites are treated using classical point charges and particle–mesh Ewald summation.^34^ A full description of the functional form and parameter choices is given in the original SWM4-NDP study.^33^ In addition to the standard LJ description, we further consider a Wang-Buckingham^29^ (WB) form for the oxygen–oxygen van der Waals interaction:

1 $$U_{\mathrm{WB}}\left(r\right)=\frac{2\epsilon }{1-\frac{3}{\gamma +3}}\frac{\sigma^{6}}{\sigma^{6}+r^{6}}\left(\frac{3}{\gamma +3}e^{\gamma \left(1-\frac{r}{\sigma }\right)}-1\right)$$

where, similar to the LJ potential, *r* is the particle pair distance, ϵ is the depth of the potential minimum, while σ sets its characteristic distance. The γ parameter is a dimensionless constant that describes the steepness of the repulsive part of the potential. Initial parameters for the WB model were obtained by fitting the WB van der Waals potential to the original LJ potential of SWM4-NDP,^35^ with the fit weighted by the pair-separation distribution sampled in molecular simulations, as given by the radial distribution function at each distance. As only oxygen–oxygen interactions are evaluated, combination rules are not applied for either functional form.

### 2.2 Bayesian Inference Framework

Parameter inference is carried out within a Bayesian framework, following exactly the same SL methodology introduced in our original study^20^ on nonpolarizable TIP3P^15^-like water models and subsequently extended to polarizable descriptions. Model parameters are treated as random variables, and uniform prior distributions encode physically reasonable bounds without enforcing overly restrictive assumptions. Given the intractability of the exact likelihood for simulation-derived observables, we employ a SL^11^ constructed from low-dimensional summary statistics obtained from molecular dynamics simulation trajectories.

The inference is constrained using a set of thermodynamic, structural, and hydrogen-bond properties, namely the enthalpy of vaporization Δ*H*_*vap*_, the molecular volume *V*_*M*_, the position and height of the first oxygen–oxygen RDF peak (*r̃*_*OO*,1_ and *g̃*_*OO*,1_), and the average hydrogen-bond donor–acceptor distance and angle (⟨*r*_*HB*_⟩ and ⟨θ_*HB*_⟩). In addition, the dielectric constant ϵ_0_, the diffusion coefficient *D*, and the constant-pressure heat capacity *C*_*p*_ are evaluated as validation properties and are not included directly in the inference.

Under the assumption that these summary statistics are asymptotically multivariate normal, as observed in related molecular simulation settings,^20^ the SL provides a consistent approximation to the true likelihood. This enables direct sampling of the posterior distribution using MH,^12^ without resorting to surrogate models or reweighting schemes. Convergence diagnostics and posterior sampling protocols follow those established in our earlier work^20^ and are not repeated here.

From the posterior samples, we extract two representative parameter sets: the posterior mean and the posterior mode. The posterior *mean*, μ_θ_, defined as the average of the sampled parameter values, summarizes the central tendency of the inferred parameter distribution and is useful for uncertainty propagation. The posterior *mode*, defined as the parameter set with the highest posterior density, represents the parameter combination that is most probable to reproduce the reference data within the chosen model and is therefore a natural point of comparison with conventional fixed-parameter force fields. Reporting both summaries provides complementary information, since the most probable parameter set need not coincide with the average of the posterior when the distribution is skewed or strongly correlated. In the following, these parameter sets are denoted LJ_*mean*_ and LJ_*mode*_ for the LJ model, and WB_*mean*_ and WB_*mode*_ for the WB model.

### 2.3 Unscented Transform

Where direct evaluation from the full posterior is computationally prohibitive, uncertainty propagation is approximated using the unscented transform (UT), which propagates the posterior mean and covariance through a nonlinear mapping using a deterministic set of so-called *sigma points* rather than full posterior sampling.^36^ As in our previous study,^20^ we employ the scaled form of the UT,^37^ in which the posterior is approximated by 2*N*_θ_ + 1 sigma points, where *N*_θ_ is the number of inferred parameters. These sigma points are centered at the posterior mean parameter vector μ_θ_ and shaped by the posterior covariance matrix Σ_θ_.

The scaled UT is parametrized by α, β, and κ. Following Julier et al., κ is used to tune higher-order moments, and for approximately Gaussian variables a useful heuristic is *N*_θ_ + κ = 3, which here gives κ = 3 – *N*_θ_.^36^ In the scaled UT, α controls the spread of the sigma points and primarily affects the higher-order terms of the approximation, while β incorporates prior distributional information into the covariance estimate; for Gaussian variables, β = 2 is recommended.^37^ As in our previous study, we again use α = 0.3, since this gave UT uncertainty estimates for the inference observables that were broadly consistent with those from full MCMC sampling while avoiding systematic underestimation.^20^

With these choices, the scaling factor is

2 $$\lambda =\alpha^{2}\left(N_{\theta }+\kappa \right)-N_{\theta }$$

and the corresponding mean and covariance weights are

3 $$W_{m}^{\left(0\right)}=\frac{\lambda }{N_{\theta }+\lambda },W_{c}^{\left(0\right)}=\frac{\lambda }{N_{\theta }+\lambda }+\left(1-\alpha^{2}+\beta \right)$$

and

4 $$W_{m}^{\left(i\right)}=W_{c}^{\left(i\right)}=\frac{1}{2\left(N_{\theta }+\lambda \right)},i=1,...,2N_{\theta }$$

To generate the sigma points, the matrix square root of (*N*_θ_ + λ)­Σ_θ_ is computed, here using a Cholesky decomposition:^20,38^

5 $$\theta^{\left(0\right)}=\mu_{\theta }$$

6 $$\theta^{\left(i\right)}=\mu_{\theta }+{\left[\sqrt{\left(N_{\theta }+\lambda \right)\Sigma_{\theta }}\right]}_{:,i},i=1,...,N_{\theta }$$

7 $$\theta^{\left(N_{\theta }+i\right)}=\mu_{\theta }-{\left[\sqrt{\left(N_{\theta }+\lambda \right)\Sigma_{\theta }}\right]}_{:,i},i=1,...,N_{\theta }$$

Because the sigma points are constructed from the full covariance matrix, they generally correspond to correlated perturbations of the parameters rather than variation of one parameter at a time.

For an observable vector **Y**(θ) evaluated at the sigma points,

8 $$Y^{\left(i\right)}=Y\left(\theta^{\left(i\right)}\right)$$

the UT approximation of the propagated mean and covariance is given by

9 $${\mathrm{\mu ̂}}_{Y}=\sum_{i=0}^{2N_{\theta }}W_{m}^{\left(i\right)}Y^{\left(i\right)}$$

10 $${\mathrm{\Sigma ̂}}_{Y}=\sum_{i=0}^{2N_{\theta }}W_{c}^{\left(i\right)}\left(Y^{\left(i\right)}-{\mathrm{\mu ̂}}_{Y}\right){\left(Y^{\left(i\right)}-{\mathrm{\mu ̂}}_{Y}\right)}^{T}$$

Each sigma-point parameter set is simulated independently, and the resulting observables are combined using the UT weights to estimate the propagated mean and covariance. This provides an efficient approximation to predictive uncertainty while retaining a direct connection to the inferred posterior distribution.^20^

### 2.4 Dimer Based Model Training

A data set consisting of water dimers at different orientations and distances was generated following an established workflow^39^ and for each of these symmetry-adapted perturbation calculations were performed.^40^ Energy components as well as the total interaction energy were computed using the Psi4 software^41^ at the SAPT2+(CCD)­δM2 level of theory as recommended by Parker et al.^42^ using the aug-cc-pvtz basis set.^43^ A cutoff of 0.05 hartree was used for the exchange energy component, that is, only dimers with exchange energy of less than 0.05 hartree were included, leaving 538 water dimers. Training of parameters for the LJ and WB was done with the Alexandria Chemistry Toolkit.^44^ The resulting parameter sets are here denoted LJ_*ACT*_ and WB_*ACT*_, respectively. As in the Bayesian inference models, the electrostatic parameters of the original SWM4-NDP model^23^ were kept unmodified. The hybrid (Genetic algorithm/MCMC) algorithm was used for training, and since there are few (two respectively three) parameters only, convergence was rapid. The parameters were trained to reproduce the total SAPT interaction energy, allowing for explicit compensation of errors between energy terms.^44–46^

### 2.5 Simulation Details

All simulations were performed using OpenMM,^47^ while structural and dynamical observables were analyzed using GROMACS,^48^ mirroring the workflow of our previous study.^20^ The primary methodological difference relative to that work is the replacement of the TIP3P^15^ water model by SWM4-NDP,^23^ which introduces two additional interaction sites per molecule and explicit polarization. In all simulations, the Drude mass was set to 0.4 amu and the maximum allowed Drude displacement to 0.02 nm. Each simulation was preceded by a short energy minimization, followed by 1 ns of NVT equilibration and 1 ns of NPT equilibration.

For the Bayesian inference simulations, a time step of 0.2 fs was used to ensure numerical stability in the presence of the Drude oscillator and the rapidly varying force-field parameters explored during inference. We employed the OpenMM implementation DrudeLangevinIntegrator, with temperatures of 298.15 K for the atomic degrees of freedom and 1.0 K for the Drude degrees of freedom, and with corresponding friction coefficients of 5 and 20 ps^–1^. Pressure was maintained at 1 atm using a Monte Carlo barostat applied every 25 steps. Electrostatic interactions were treated using PME^34,49^ with a real-space cutoff of 0.8 nm and an Ewald error tolerance of 10^–4^. LJ interactions were treated with the same cutoff and analytical long-range dispersion corrections to the energy.^50^ The relatively short cutoff was chosen because some parameter combinations sampled during MCMC produced dense configurations and correspondingly small simulation boxes; using a cutoff well below half the box length helped avoid violations of the minimum-image convention and thereby prevented simulation instabilities.

For the production simulations used to evaluate dynamic properties such as the diffusion coefficient and the dielectric constant, we employed the OpenMM implementation DrudeNoseHooverIntegrator, i.e., a Drude-specific Nosé–Hoover integrator,^51,52^ using a chain length of 3, 3 time steps in the multiple-time-step scheme,^53^ and 3 terms in the Yoshida-Suzuki decomposition.^54,55^ The atomic and Drude degrees of freedom were thermostated at 298.15 and 1.0 K, respectively, and pressure was maintained at 1 atm using a Monte Carlo barostat applied every 100 steps. Electrostatic interactions were again treated using PME,^34,49^ here with a real-space cutoff of 0.9 nm and an Ewald error tolerance of 10^–4^, while LJ interactions were treated with a cutoff of 0.9 nm and analytical long-range dispersion corrections to the energy.^50^ We also tested a longer time step, but this proved unstable for some LJ sigma-point parameter sets and for most Wang-Buckingham sigma-point parameter sets. Therefore, despite the models being rigid,^56^ we retained a time step of 0.2 fs in the production simulations. Each production simulation consisted of 50 ns, and these trajectories were divided into five 10 ns blocks for uncertainty estimation.

Thermodynamic and structural observables were evaluated at ambient conditions using the same definitions, estimators, and statistical treatment as in the original protocol.^20^ Two exceptions concern the estimation of the constant-pressure heat capacity *C*_*p*_ and the diffusion coefficient *D*. Because OpenMM does not record instantaneous pressure when using a Monte Carlo barostat,^57^ and because GROMACS does not readily support the WB functional form, *C*_*p*_ was estimated from the finite-difference slope of the enthalpy evaluated at temperatures of ± 5 K around the reference temperature of 298.15 K using OpenMM. This procedure replaces the fluctuation-based estimator used previously but remains consistent with the underlying statistical framework. Diffusion coefficients were estimated from the mean square displacement, and the resulting values were corrected for finite box-size effects using the expression of Yeh and Hummer:^58^

11 $$D=D_{\mathrm{PBC}}+\frac{k_{B}T\xi }{6\pi \eta L}$$

where *k*_*B*_ is the Boltzmann constant, *T* the absolute temperature, ξ a constant 2.837297,^58^ *L* the box edge and η the viscosity. For the latter, we used the published value of 0.66 cP for SWM4-NDP^27^ for all models. Furthermore, a quantum correction term for *C*_*V*_ at 300 K, taken from Waheed and Edholm,^59^ was applied when estimating *C*_*p*_.

### 2.6 Uncertainty Quantification

Posterior samples obtained from the Bayesian inference were propagated to observables of interest to quantify both parameter uncertainty and predictive variability. As in our earlier work, we distinguish between uncertainty arising from the inferred parameter distributions, residual model bias associated with the chosen functional form, and finite sampling effects. These contributions are quantified using the coefficient of variation (CV), relative bias (RB), and relative standard error (RSE), respectively. These properties are given by

12 $$\mathrm{CV}\left(Y\right)=\frac{\sqrt{\mathrm{Var}\left(Y\right)}}{E\left[Y\right]}$$

where Var indicates the variance of a simulated observable *Y*, and E the expected value of the same observable,

13 $$\mathrm{RB}\left(Y,y\right)=\frac{y-E\left[Y\right]}{E\left[Y\right]}$$

for an experimental reference value *y*. With this definition, a positive RB indicates that the experimental reference exceeds the predicted mean, that is, the model underestimates the observable, whereas a negative RB indicates overestimation. Finally,

14 $$\mathrm{RSE}\left(Y\right)=\frac{s_{B}/\sqrt{N_{B}}}{E\left[Y\right]}$$

indicating a block-averaging procedure was used consisting of *N*_*B*_ blocks with standard deviation *s*_*B*_.

## 3 Results and Discussion

In what follows, we first describe the Bayesian parameter inference, then compare the results to those from the gas-phase training and summarize the results in concluding section.

### 3.1 Bayesian Inference

After removing the burn-in of the MCMC chains corresponding to the LJ model variant, 87,382 posterior samples remained, each corresponding to an accepted parameter set evaluated via a 100 ps molecular simulation, and the resulting parameter posterior is shown in Figure 1. Due to how the LJ potential is defined, there is inevitably an anticorrelation between ϵ and σ. The posterior appears to shift the original SWM4-NDP model toward lower ϵ and higher σ, but the original model still falls well within the high-posterior-density ridge associated with this anticorrelation, albeit in a region of lower posterior density. The (ϵ, σ) posterior is quite similar to the posterior for three-point water models of our previous work^20^ (Figure S15 in that paper), the major difference being that the ϵ posterior allows for higher values for polarizable water. Because the original SWM4-NDP model already has a higher ϵ value than the original TIP3P model, the shift of the posterior toward somewhat higher ϵ values for polarizable water is expected and does not represent a substantial difference between the two posterior distributions.

**Figure 1:**
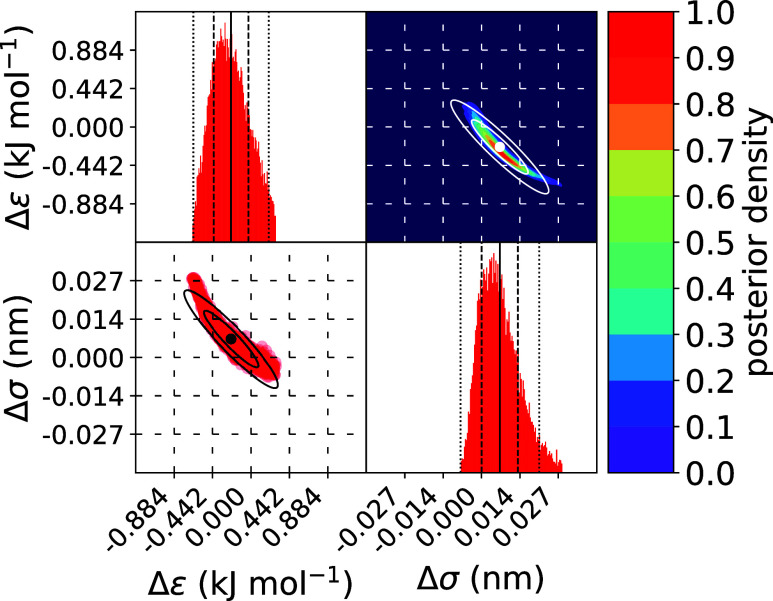
Posterior deviations of Lennard-Jones 12–6 parameters relative to the SWM4-NDP reference. Shown are differences between (ϵ, σ) samples drawn from the posterior and the corresponding Lennard-Jones 12–6 parameters of the SWM4-NDP model. Each panel is centered such that zero denotes agreement with the reference parametrization. Marginal posterior distributions are shown along the diagonal, while off-diagonal panels illustrate joint parameter distributions, represented as scaled kernel density estimates in the upper triangle and individual samples in the lower triangle. Solid lines indicate posterior means; dashed lines mark the central 64.2% and 95.4% credibility intervals. The same symbols are used to denote the corresponding confidence regions in the two-dimensional projections. Note that *y*-axis scaling applies only to the off-diagonal panels.

The observables in Figure 2 corresponding to the parameter posterior in Figure 1, display similarities to Figure S16 of our earlier work.^20^ The most notable difference here is that ⟨θ_HB_⟩ of TIP3P is beyond two standard deviations away from the experimental reference while SWM4-NDP is barely one standard deviation away, indicating a more physically realistic description of hydrogen-bond geometry in SWM4-NDP.

**Figure 2:**
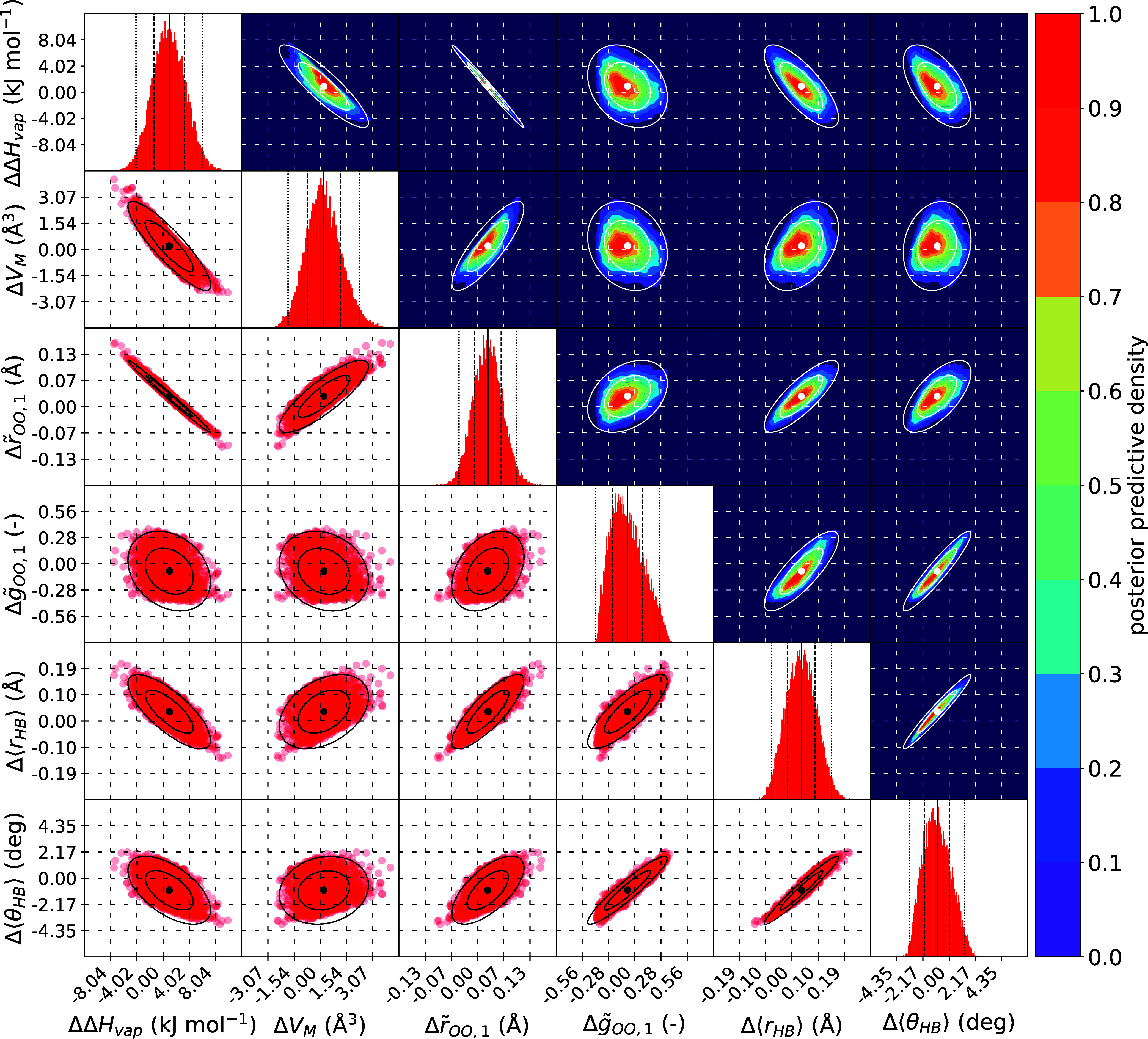
Posterior distributions of observable deviations from experiment for the Lennard-Jones 12–6 model. Differences between simulated observables and their experimental reference values are shown for parameter samples drawn from the (ϵ, σ) posterior of the Lennard-Jones 12–6 interaction. All panels are centered at zero, corresponding to perfect agreement with the experimental reference. Marginal distributions of each observable are shown on the diagonal, while pairwise correlations are visualized off-diagonal using scaled kernel density estimates (upper triangle) and individual posterior samples (lower triangle). Solid lines denote posterior means, with dashed lines indicating the central 64.2% and 95.4% credibility intervals. Matching markers and contours highlight the corresponding regions in the two-dimensional projections. The *y*-axis labels apply only to off-diagonal panels.

For the WB model variant, 73,997 posterior samples remained after removal of the burn-in period, and the resulting posterior is shown in Figure 3. The posterior mean favors lower ϵ and higher σ and γ relative to the original parameters. Unlike the results for LJ (Figure 1), there is no longer a clear correlation between ϵ and σ; instead, the posterior structure reflects a trade-off between ϵ and γ, while variations in σ play a secondary role. The lower bound in ϵ is imposed by the prior to prevent the van der Waals interaction from vanishing, which would lead to unstable simulations. Likewise, large γ values are suppressed by the prior, as further increases do not materially alter the potential due to rapid convergence of WB behavior. The predicted observables corresponding to the WB parameter posterior in Figure 3 are presented in Figure 4.

**Figure 3:**
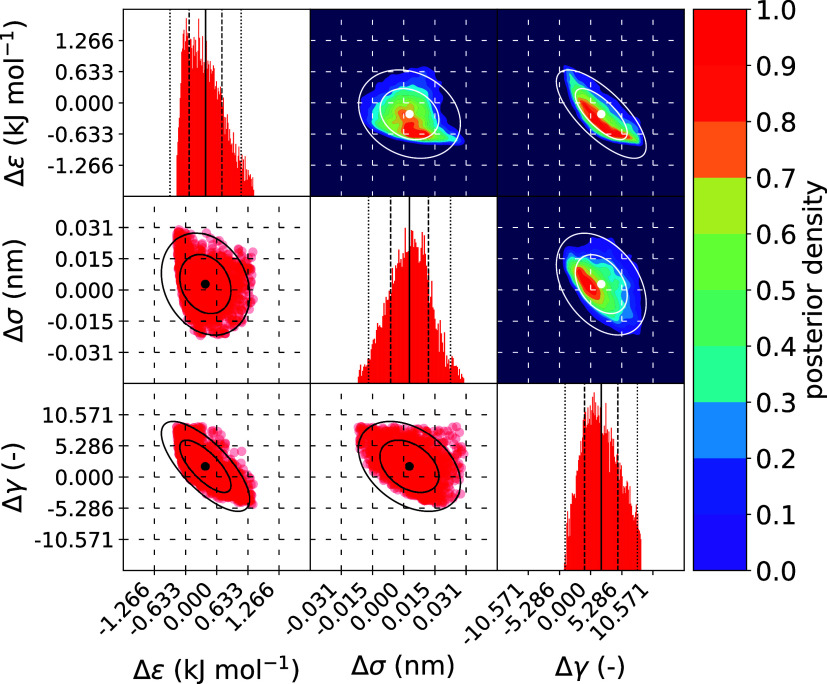
Posterior deviations of Wang-Buckingham parameters relative to the modified SWM4-NDP reference. Shown are differences between (ϵ, σ, γ) samples drawn from the posterior and the corresponding Wang-Buckingham parameters of the modified SWM4-NDP model. Each panel is centered such that zero denotes agreement with the reference parametrization. Marginal posterior distributions are shown along the diagonal, while off-diagonal panels illustrate joint parameter distributions, represented as scaled kernel density estimates in the upper triangle and individual samples in the lower triangle. Solid lines indicate posterior means; dashed lines mark the central 64.2 and 95.4% credibility intervals. The same symbols are used to denote the corresponding confidence regions in the two-dimensional projections. Note that *y*-axis scaling applies only to the off-diagonal panels.

**Figure 4:**
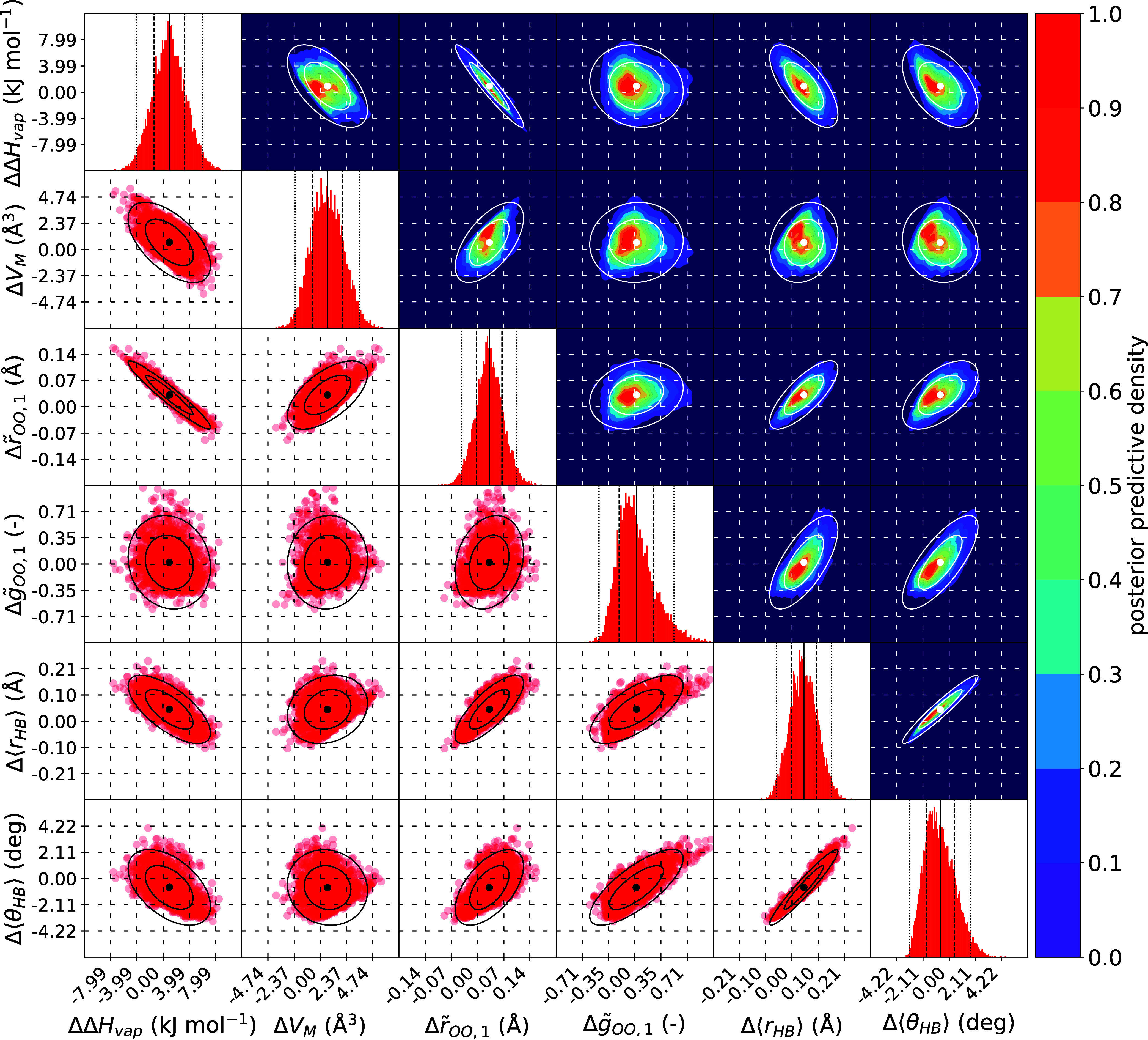
Posterior distributions of observable deviations from experiment for the Wang-Buckingham model. Differences between simulated observables and their experimental reference values are shown for parameter samples drawn from the (ϵ, σ, γ) posterior of the Wang-Buckingham interaction. All panels are centered at zero, corresponding to perfect agreement with the experimental reference. Marginal distributions of each observable are shown on the diagonal, while pairwise correlations are visualized off-diagonal using scaled kernel density estimates (upper triangle) and individual posterior samples (lower triangle). Solid lines denote posterior means, with dashed lines indicating the central 64.2 and 95.4% credibility intervals. Matching markers and contours highlight the corresponding regions in the two-dimensional projections. The *y*-axis labels apply only to off-diagonal panels.

The distributions in Figure 4 show a larger variance than that of those in Figure 2. Table 1 provides a numeric comparison of the models. The CVs from MCMC show an equal or higher variance for all properties except Δ*H*_vap_ for WB as compared to LJ. However, in all cases the order of magnitude is similar between models and, in addition, the CVs of LJ for SWM4-NDP are of comparable magnitude to that for TIP3P.^20^ This suggests that, for the present observable set, the overall pattern of propagated parameter uncertainty depends more on the observable being considered than on the underlying model class. In other words, the CV appears to reflect primarily how strongly a given observable responds to changes in the van der Waals parameters and how tightly those parameter directions are constrained by the reference data. Differences between LJ and WB are still present, but they act mainly as a secondary modulation of this observable-dependent pattern. As for the nonpolarizable model, the RSEs are negligible for the inference properties.

**Table 1:** Relative Simulation Errors for Selected Observables *Y* Obtained Using Different van der Waals Functional Forms^a^

				CV(*Y*) (%)		RB(*Y*, *y*) (%)		RSE(*Y*) (%)	
property	*y*	refs	vdW	θ_MCMC_	θ_UT_	θ_MCMC_	θ_UT_	θ_UT_	μ_θ_
Δ*H*_vap_	44.0 $\frac{\mathrm{kJ}}{\mathrm{mol}}$	^60^	LJ	5.7	11	–2.1	–1.6	0.0	0.0
			WB	5.6	23	–2.1	0.3	0.0	0.0
*V*_*M*_	30.0 Å^3^	^60^	LJ	3.5	6.9	–0.7	–1.1	0.0	0.0
			WB	4.8	15	–2.1	–4.1	0.0	0.0
*r̃*_*OO*,1_	2.97 Å	^61^	LJ	1.2	2.3	–0.9	–1.0	0.0	0.0
			WB	1.2	4.8	–1.0	–1.6	0.0	0.0
*g̃*_*OO*,1_	2.14	^61^	LJ	8.3	8.6	3.8	3.8	0.2	0.0
			WB	12	16	–1.1	–1.8	0.3	0.0
⟨*r*_HB_⟩	1.93 Å	^62^	LJ	2.8	4.0	–1.7	–1.9	0.0	0.0
			WB	2.8	8.0	–2.4	–3.3	0.1	0.0
⟨θ_HB_⟩	14.7 deg	^62^	LJ	8.3	9.9	7.3	6.9	0.2	0.0
			WB	8.8	18	5.1	3.1	0.2	0.0
ϵ_0_	78.4	^60^	LJ		15		11	15	3.2
			WB		48		–7.9	23	1.4
*D*	2.30 × 10^–5^ $\frac{{\mathrm{cm}}^{2}}{s}$	^63^	LJ		110^b^		31^b^	13^b^	1.3^b^
			WB		180^b^		–6.2^b^	16^b^	1.1^b^
*C*_*p*_	75.3 $\frac{J}{\mathrm{molK}}$	^60^	LJ		75^c^		–15^c^	0.0^c^	0.0^c^
			WB		20^c^		–13^b^	0.0^c^	0.0^c^

a Reported metrics include the coefficient of variation CV­(*Y*), relative bias RB­(*Y*, *y*) with respect to experimental reference values *y*, and relative standard error RSE­(*Y*). For each observable, results are shown separately for the Lennard-Jones (LJ) and Wang-Buckingham (WB) models, as indicated in the *vdW* column. The observables *Y*(*θ*) are evaluated using parameter samples *θ*_MCMC_ drawn from the posterior, unscented transform sigma points *θ*_UT_ constructed from the posterior mean and covariance. The RSE for the posterior mean parameter set *μ*_*θ*_ is reported separately to show the simulation error of a single simulation, unaffected by unscented-transform weighting. Reference values *y* and corresponding sources are listed.

b Includes correction according to Yeh and Hummer^58^ with viscosity from Teng and co-workers.^27^

c Includes correction from Waheed and Edholm.^59^

The increased posterior variance observed for the WB model relative to the LJ model is nevertheless expected, as changes in ϵ and σ affect both van der Waals functional forms in broadly similar ways, while the additional γ parameter gives the WB model greater flexibility by increasing the number of parameter combinations that can reproduce the reference data. Taken together, these results suggest that the observable under consideration primarily determines how much uncertainty is seen in the predicted observables, whereas the choice of functional form mainly affects the magnitude of that uncertainty.

A key observation is that the CVs of the inferred van der Waals parameter posterior remain comparable to that obtained previously for TIP3P-like models, despite the inclusion of explicit polarization. This indicates that, for the present set of observables, the degree to which short-range nonbonded parameters are constrained by the reference data is similar across these model classes. More generally, the CVs reflect how effectively the chosen observables constrain the parameters within a given functional form and parametrization space. In contrast, the RBs are clearly functional-form dependent, revealing systematic deviations that persist across the posterior even when parameters are well constrained. While polarization reduces bias for certain observables, others remain unaffected, emphasizing that the RB statistic is governed primarily by limitations of the functional form rather than by parameter uncertainty.

### 3.2 Analysis of Uncertainties

The UT sigma-point uncertainty estimates (see Section 2) obtained for the polarizable SWM4-NDP model show trends similar to those observed in our TIP3P study.^20^ In particular, the UT tends to slightly overestimate the CV for the inference observables, which is consistent with our choice of α = 0.3 to avoid systematic underestimation. For the diffusion coefficient *D*, the main issue is the UT estimate of uncertainty rather than the point prediction itself. The UT-based CV is 110% for LJ and 180% for WB (Table 1), compared to 38% for TIP3P with LJ in our earlier study.^20^ This reflects a limitation of the UT approximation for *D*: the uncertainty propagated from the posterior mean and covariance through the sigma points becomes so large that the implied uncertainty range extends into negative diffusivities, which is not physically meaningful. For this observable, the large CV should therefore be interpreted mainly as a sign that the UT performs poorly, particularly for WB, rather than as a quantitatively reliable uncertainty estimate. By contrast, the RB of *D* is smaller in magnitude in the present study, with values of +31% for LJ and −6.2% for WB, compared to −63% for TIP3P with LJ.^20^ Part of this difference is due to the Yeh-Hummer correction in eq 11, which was applied here but not in the earlier TIP3P analysis; applying the same correction there would shift the TIP3P value only modestly, to approximately −53%, so the improvement in RB is not explained by the correction alone.

For the dielectric constant ϵ_0_, the LJ functional form gives a smaller RB, whereas the larger coefficient of variation associated with the WB form suggests a higher probability that the experimental reference falls within the predicted uncertainty range. The smaller magnitude of the RB for *C*_*p*_ in the present SWM4-NDP study is mainly due to the applied correction. For LJ, the RB is −15 here, compared to −44 in our earlier TIP3P study; applying the same correction to TIP3P would reduce this to approximately −25, so the present improvement would still be only partial.

### 3.3 Models Trained on Gas-Phase Data

Table 2 shows the correlation between models trained on dimer energies and the reference data from SAPT. In addition, correlation for the original SWM4-NDP model, and the LJ_*mode*_ and WB_*mode*_ models are given. Although the differences are small, the WB_*ACT*_ has somewhat lower root-mean-square deviation from the SAPT reference than the other models. The resulting dimer potentials are plotted in Figure 5. Here too, the WB_*ACT*_ is the outlier, being clearly less repulsive at short oxygen–oxygen distance, and somewhat more attractive at distances ranging from 0.35 to 0.5 nm as well. The steepness of the potential is governed by the γ parameter which is considerably smaller for WB_*ACT*_ than for WB_*mode*_ derived from Bayesian inference.

**Figure 5:**
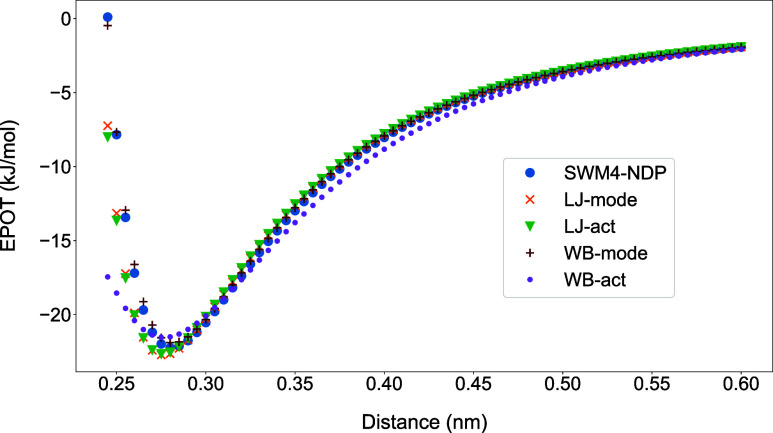
Potential energy of water dimers as a function of distance for five water models. At each oxygen–oxygen distance (*x*-axis), the position of the hydrogen atoms was minimized and the resulting potential energy plotted. Figure produced using plotXVG.^78^

**Table 2:** Root Mean Square Deviation (RMSD) from the Total SAPT Interaction Energy and Pearson Correlation Coefficient *r* ^2,^ ^a^

model	RMSD (kJ/mol)	r^2^
SWM4-NDP	2.70	0.86
LJ_mode_	2.55	0.88
LJ_ACT_	2.54	0.88
WB_mode_	2.75	0.96
WB_ACT_	2.35	0.89

a Data is shown for the *mode* water models from Bayesian inference (those that are most likely to reproduce experimental reference data from the liquid phase) and for the *act* models trained on SAPT interaction energies. Statistics over 538 dimers.

The WB_*ACT*_ reproduces the position and depth of the energy minimum best of the models evaluated (Figure 5), but as a result, liquid phase simulations are unstable (Table 3). The WB^29^ potential was designed to circumvent the singularity at short distance that occurs with the traditional Buckingham potential.^64^ In fact, quite a few potentials have been proposed to remedy this problem^65–67^ and these potentials have been evaluated extensively on noble gases.^68–71^ However, when combining such potentials with point-charge Coulomb interactions, a new singularity is created leading to unstable simulation if the repulsion is too weak, as for WB_*ACT*_. Even apart from the fact that simple point charges are a poor approximation of atomic charge distributions,^72^ the combination of WB with point charges can lead to unstable simulations. Using Gaussian- or Slater-distributed charges^73^ will resolve this problem, as was demonstrated with a phase-transferable model for alkali-halides.^30,74–77^

**Table 3:** Simulated Observables for the SWM4-NDP Reference Model Using Lennard-Jones (LJ) and Wang-Buckingham (WB) van der Waals Interactions^a^

	Δ*H*_vap_	*V*_*M*_	*r̃*_*OO*,1_	*g̃*_*OO*,1_	⟨*r*_HB_⟩	⟨θ_HB_⟩	ϵ_0_	*D*	*C*_*p*_	MAPE
model	( $\frac{\mathrm{kJ}}{\mathrm{mol}}$ )	(Å^3^)	(Å)	(−)	(Å)	(deg)	(−)	(10^–5^ $\frac{{\mathrm{cm}}^{2}}{s}$ )	( $\frac{J}{\mathrm{molK}}$ )	(%)
Exp	44.0	30.0	2.97	2.14	1.93	14.7	78.4	2.30	75.3	-
LJ	44.0	30.0	3.01	2.25	2.01	14.9	77.3	2.74^b^	79.6^c^	4.25
LJ_mean_	42.3	31.2	3.03	2.08	2.00	14.2	72.5	2.64^b^	85.6^c^	6.19
LJ_mode_	44.5	30.5	3.00	2.00	1.95	13.4	77.8	1.67^b^	91.8^c^	7.81
LJ_ACT_	44.1	30.1	3.00	1.94	1.94	13.1	80.3	1.62^b^	95.4^c^	9.00
WB	43.8	30.1	3.01	2.25	2.01	14.9	76.0	2.71^b^	79.5^c^	4.36
WB_mean_	38.2	33.4	3.10	2.33	2.08	15.7	60.1	4.52^b^	77.5^c^	19.5
WB_mode_	42.0	31.3	3.04	2.19	2.02	14.7	71.4	2.96^b^	81.5^c^	7.12
WB_ACT_	N/A	N/A	N/A	N/A	N/A	N/A	N/A	N/A	N/A	N/A

a Experimental reference values are listed in the first data row. Values are also shown for posterior summary estimates (posterior mean and posterior mode). Uncertainty estimates for these properties, including the coefficient of variation, relative bias, and simulation error, are reported separately in Table 1. The mean average percent error (MAPE) is given in the last column. Simulations reported in this table were performed with fixed parameters.

b Includes correction according to Yeh and Hummer^58^ with viscosity from Teng and co-workers.^27^

c Includes correction from Waheed and Edholm.^59^

### 3.4 Comparison of Models

From the MCMC sampling, it is evident that the two van der Waals variants prefer a significantly smaller ϵ compared to the original model, for both the *mean* and *mode* (see Section 2) of the distributions (Table 4). The SAPT training follows the same trend for LJ but the complete opposite can be observed for WB: for WB_*ACT*_, ϵ is twice the original value whereas γ is 25% lower. This observation is consistent with the strong correlation between ϵ and γ found in MCMC (Figure 3). In contrast, higher σ values are preferred for both variants, for MCMC as well as ACT training. It should be emphasized, however, that parameters should not be interpreted in isolation, as their correlations are complex (Figures 1 and 3).

**Table 4:** Model Parameters for the SWM4-NDP Reference Model Using Lennard-Jones (LJ) and Wang-Buckingham (WB) van der Waals Interactions^a^

	ϵ	σ	γ
Model	( $\frac{\mathrm{kJ}}{\mathrm{mol}}$ )	(nm)	(−)
LJ	0.882573	0.318395	
LJ_mean_	0.652832	0.324873	
LJ_mode_	0.570174	0.325996	
LJ_ACT_	0.492896	0.329265	
WB	0.845760	0.357522	17.5048
WB_mean_	0.617963	0.360377	19.3043
WB_mode_	0.698808	0.361097	17.8118
WB_ACT_	1.746519	0.362989	13.0751

a Values are shown for the original reference parameterization and for posterior summary estimates (posterior mean and posterior mode).

The reference models corresponding to the two van der Waals potentials exhibit similar levels of accuracy, as reflected in their simulated means across observables in Table 3, validating the conversion from the LJ potential to the WB form (see Section 2). Moreover, both reference models outperform the Bayesian MCMC and ACT-based models. This is consistent with the role of Bayesian inference, which is designed to characterize the full parameter posterior rather than to identify a single optimal parameter set. Since all observables are considered simultaneously, their relative weighting influences how well different regions of parameter space explain the data. Summary statistics such as the posterior mean or mode therefore need not correspond to the narrow, lower-probability regions that yield the best overall agreement. In addition, the ACT SAPT parametrization was trained on gas-phase dimers rather than condensed-phase systems, which further explains its reduced performance for bulk properties.

Another counterintuitive outcome of UT is the apparent overestimation of *D* in Table 3 for WB_mean_, whereas the RB (eq 13) reported in Table 1 suggests that *D* is barely overestimated at all. This discrepancy arises because, although the posterior mean corresponds to a relatively high value of *D*, the surrounding region of parameter space, captured via the sigma-point approximation, estimates substantially lower diffusivities as their weights are considered (see Table S2). As a result, the UT-based prediction of *D* is shifted toward lower values rather than higher ones.

It is interesting as well to compare the LJ_ACT_ model to LJ_mode_. Their parameters are very similar in Table 4 and as a result, the properties predicted by both models are also very similar (Table 3). The training of the WB_ACT_ model, on the other hand, did not yield a stable model for bulk simulation, which could be explained by a mix between the significantly higher ϵ and σ and lower γ compared to the other models listed in Table 4, which suggests the Coulomb singularity is accessible during MD simulations.

### 3.5 Relation between Observables and Parameters

During MCMC in parameter space, the parameters as well as all six observables used in inference were saved. This makes it possible to plot how each observable deviates from experiment across parameter space. Figure 6 shows, for the LJ sampling, the median relative deviation from experiment for each observable as a function of (ϵ, σ). These heatmaps are purely descriptive: they show closeness to experiment at the level of the median, but do not account for the variance and covariance that enter the likelihood. A smaller percent deviation in the figure therefore does not necessarily correspond to a higher posterior density. Nevertheless, the figure clearly shows that closest agreement with experiment for the different observables requires different (ϵ, σ) combinations. In particular, *g*_*OO*,1_ and ⟨θ_*HB*_⟩ yield reasonable agreement with experiment only for intermediate values of ϵ, and the shapes of the dark bands differ between observables. These findings reiterate that the observables evaluated here place different, and in part incompatible, constraints on the parameters. This result can be compared to our earlier work on nonpolarizable TIP3P, where we performed inference calculations using either Δ*H*_vap_ and *V*_*M*_, *r̃*_*OO*,1_ and *g̃*_*OO*,1_, or ⟨*r*_HB_⟩ and ⟨θ_HB_⟩, and found significantly different parameter distributions that barely overlapped.^20^

**Figure 6:**
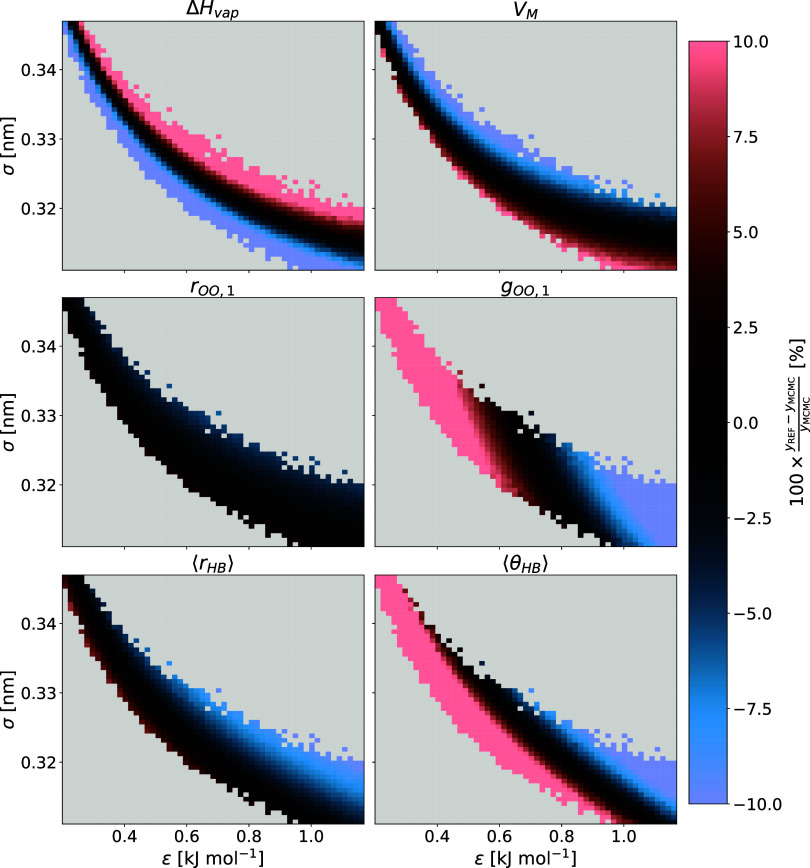
Median relative deviations from experiment for the six observables used in the Lennard-Jones 12–6 inference, shown across the sampled (ϵ, σ) parameter space. Each panel reports the percent deviation from the experimental reference for one observable. The distinct low-deviation bands reveal that different observables are best reproduced in different regions of parameter space, highlighting the competing constraints they impose on the model.

Figures S1–S3 show plots akin to Figure 6 for the WB potential sampling. Since there are three parameters, the observable deviations are plotted for (ϵ, σ), (ϵ, γ), and (σ, γ) combinations separately. The plots are somewhat more noisy due to sampling in three dimensions but, like for the LJ sampling (Figure 6), the *g*_OO,1_ and ⟨θ_HB_⟩ differ most from the other observables. Since both LJ and WB yield qualitatively similar plots, the overall parameter dependence of the six observables appears to be similar for the two functional forms. However, the relative biases show that the precise choice of van der Waals functional form still matters quantitatively, as LJ and WB reproduce different observables with different accuracy.

### 3.6 Lessons for Force Field Development

The two van der Waals functional forms differ in which structural and hydrogen-bond observables they reproduce most accurately. The LJ potential yields lower RB for the RDF peak position *r̃*_*OO*,1_ and the average hydrogen-bond distance ⟨*r*_HB_⟩, whereas the WB potential performs better for the RDF peak height *g̃*_*OO*,1_ and the hydrogen-bond angle ⟨θ_HB_⟩. This indicates that the functional forms capture complementary features of liquid structure and hydrogen bonding, arising from observable-specific sensitivities and parameter compensation within the likelihood function.

The observations made here resembles the work by Teng et al. on variants of the SWM4-NDP model, notably SWM4-HLJ that includes a LJ interaction on the hydrogen atoms as well.^27^ For the models evaluated in that paper, polarizable models are introduced that either have an enhanced dipole compared to experiment, have LJ interactions on the hydrogen atoms, or both. These models are then compared to SWM4-NDP^23^ as well as the six-point SWM6 ^24^ and AMOEBA water.^79^ Interestingly, the model SWM4-HD in that paper^27^ with an enhanced dipole and reduced polarizability compared to experiment, performs better for cluster energies whereas some other models perform better in the liquid phase. Taken together with the results in our analyses, these issues suggest that the models studied here, as well as by Teng and co-workers^27^ still are compromises hampered by lacking elements of the physics needed to described both the gas-phase and condensed phases.^7^ An important observation, made by a number of authors, is that polarizability is not a constant number, in particular at short distance,^80–82^ making it even more difficult to derive force field models including polarization from dimer data.^40^ Nevertheless, the close agreement between the LJ van der Waals parameters obtained from Bayesian posterior modes, LJ_mode_, and those derived independently from SAPT dimer training, LJ_ACT_, is an encouraging result. It suggests that gas-phase dimer energetics contain information that is directly relevant for describing the liquid phase and that phase-transferable van der Waals parameters may, in some cases, be obtained from gas-phase data alone.

## 4 Conclusion

The Bayesian framework used here is not intended as a model optimization tool, but as a means of sampling and analyzing the posterior associated with a fixed functional form. Accordingly, CV and RB should not be interpreted as measures of model quality in isolation. A large CV indicates that the available observables do not sufficiently constrain certain parameters, suggesting that additional or alternative reference data may be required for meaningful parametrization. A large RB, on the other hand, signals a limitation of the adopted functional form or fixed model assumptions, for which further parametrization within the same model class is unlikely to yield improvement. Together, CV and RB provide complementary diagnostics that distinguish between under-constrained parameters and intrinsic model inadequacy. The Bayesian framework is particularly useful here because access to the full posterior makes it possible to separate uncertainty arising from limited parameter identifiability from systematic errors that persist across plausible parameter sets. This, in turn, helps indicate whether future effort should focus on improved parametrization, on revisiting the underlying functional form, or on both.

Although the inclusion of explicit polarization in SWM4-NDP reduces the bias of ⟨θ_HB_⟩ relative to nonpolarizable three-point models, substantial deviation from experiment remain in the models studied here as well as in similar models from the literature.^21–23,27,79^ This mirrors our earlier findings for TIP3P-like models, where ⟨θ_HB_⟩ was shown to be only weakly sensitive to nonbonded parameter tuning despite well-constrained posteriors. Together, these results reinforce that the dominant contributions to hydrogen-bond structure and energetics arise from molecular geometry and charge placement, which define both the directional electrostatics and the underlying hydrogen-bond network. In this context, van der Waals interactions primarily provide fine-tuning rather than control of hydrogen-bond structure. Consequently, while polarization alleviates some systematic bias, further reductions of the RB, particularly for angular hydrogen-bond metrics, are likely to require modifications to molecular geometry and electrostatics rather than additional refinement of short-range van der Waals parameters. In sum, the present analysis not only identifies the limitations of the current models but also provides a principled basis for developing more accurate and physically complete force fields.

## Supplementary Material



## Data Availability

All code and data used here are available from https://github.com/AlexandriaChemistry/Data

## Notes

Additional figures corresponding to the relation between force field parameters and observables, and tables with results related to uncertainty estimates (PDF)
