# Supplementary material for: Bayesian Modeling of Polarizable Water: Lessons for Force Field Development
Source: J Chem Theory Comput. 2026 May 23;22(11):5435–47. doi: 10.1021/acs.jctc.6c00260 (PMC13255248; doi:10.1021/acs.jctc.6c00260)
Supplement: Supplementary file 1 [file ct6c00260_si_001.pdf]

Supporting Information for "Bayesian Modeling of Polarizable Water - Lessons for Force Field Development"

Alfred T. Nordman,[†] Stefan Engblom,[‡,¶] and David van der Spoel[*,†]

†*Department of Cell and Molecular Biology, Uppsala University, Uppsala, Sweden*

‡*Division of Scientific Computing, Department of Information Technology, Uppsala University, Uppsala, Sweden*

¶*Science for Life Laboratory, Department of Information Technology, Uppsala University, Uppsala, Sweden*

E-mail: david.vanderspoel@icm.uu.se

Contents

1 Sigma-point data **S2**

2 Parameter pairs and corresponding observables **S4**

References **S7**

1 Sigma-point data

Table S1: Parameters, simulated observables, and unscented-transform weights for the Lennard-Jones 12-6 sigma points constructed from the posterior mean and covariance. The table includes the posterior mean parameter set, the four off-center sigma points, the corresponding observables, and the experimental reference values.

Model	Exp	LJ_{mean}	LJ_{mean-1}	LJ_{mean-2}	LJ_{mean+1}	LJ_{mean+2}
Parameter						
ϵ ($\frac{kJ}{mol}$)	–	0.652832	0.539957	0.652832	0.765706	0.652832
σ (nm)	–	0.324873	0.328270	0.323586	0.321476	0.326161
Observable						
ΔH_{vap} ($\frac{kJ}{mol}$)	44.0	42.3	42.7	44.2	43.1	40.6
V_M (Å^3)	30.0	31.2	31.3	30.5	30.6	32.0
$\tilde{r}_{OO,1}$ (Å)	2.97	3.03	3.02	3.00	3.02	3.06
$\tilde{g}_{OO,1}$ (–)	2.14	2.08	1.99	2.06	2.17	2.10
$\langle r_{HB} \rangle$ (Å)	1.93	2.00	1.97	1.97	2.00	2.02
$\langle \theta_{HB} \rangle$ (deg)	14.7	14.2	13.6	13.8	14.6	14.6
ϵ_0 (–)	78.4	72.5	74.2	78.8	74.2	68.1
D (10^{-5} $\frac{cm^2}{s}$)	2.30	2.65*	2.11*	2.05*	2.69*	3.28*
C_p ($\frac{J}{mol\,K}$)	75.3	85.6**	90.8**	88.0**	82.2**	82.8**
Unscented transform weights						
Mean W_m	–	$-6.\overline{407}$	$1.\overline{851}$	$1.\overline{851}$	$1.\overline{851}$	$1.\overline{851}$
Variance W_c	–	$-3.49\overline{740}$	$1.\overline{851}$	$1.\overline{851}$	$1.\overline{851}$	$1.\overline{851}$

* Includes correction according to Yeh and Hummer[1] with viscosity from Teng and coworkers.[2]

** Includes correction from Waheed and Edholm.[3]

Table S2: Parameters, simulated observables, and unscented-transform weights for the Wang-Buckingham sigma points constructed from the posterior mean and covariance. The table includes the posterior mean parameter set, the six off-center sigma points, the corresponding observables, and the experimental reference values.

Model	Exp	WB_{mean}	WB_{mean-1}	WB_{mean-2}	WB_{mean-3}	WB_{mean+1}	WB_{mean+2}	WB_{mean-3}
Parameter								
ϵ ($\frac{kJ}{mol}$)	–	0.617963	0.430086	0.617963	0.617963	0.805840	0.617963	0.617963
σ (nm)	–	0.360377	0.361500	0.355274	0.360377	0.359254	0.365479	0.360377
γ (-)	–	19.3043	20.4754	20.1793	18.6637	18.1333	18.4294	19.9450
Observable								
ΔH_{vap} ($\frac{kJ}{mol}$)	44.0	38.2	38.7	40.1	41.0	39.8	36.8	36.0
V_M (Å^3)	30.0	33.4	33.9	32.6	32.0	32.0	34.1	34.7
$\tilde{r}_{OO,1}$ (Å)	2.97	3.10	3.09	3.07	3.05	3.07	3.12	3.13
$\tilde{g}_{OO,1}$ (–)	2.14	2.33	2.25	2.41	2.23	2.36	2.24	2.41
$\langle r_{HB} \rangle$ (Å)	1.93	2.08	2.06	2.06	2.03	2.07	2.09	2.12
$\langle \theta_{HB} \rangle$ (deg)	14.7	15.7	15.1	15.6	14.9	15.8	15.7	16.3
ϵ_0 (–)	78.4	60.1	60.2	63.4	69.4	64.8	58.2	56.4
D (10^{-5} $\frac{cm^2}{s}$)	2.30	4.52*	3.61*	3.88*	3.25*	4.26*	5.19*	6.18*
C_p ($\frac{J}{mol\,K}$)	75.3	77.5**	83.5**	78.2**	81.7**	74.7**	77.1**	74.5**
Unscented transform weights								
W_m	–	$-10.\overline{1}$	$1.\overline{851}$	$1.\overline{851}$	$1.\overline{851}$	$1.\overline{851}$	$1.\overline{851}$	$1.\overline{851}$
W_c	–	$-7.20\overline{1}$	$1.\overline{851}$	$1.\overline{851}$	$1.\overline{851}$	$1.\overline{851}$	$1.\overline{851}$	$1.\overline{851}$

* Includes correction according to Yeh and Hummer[1] with viscosity from Teng and coworkers.[2]
** Includes correction from Waheed and Edholm.[3]

2 Parameter pairs and corresponding observables

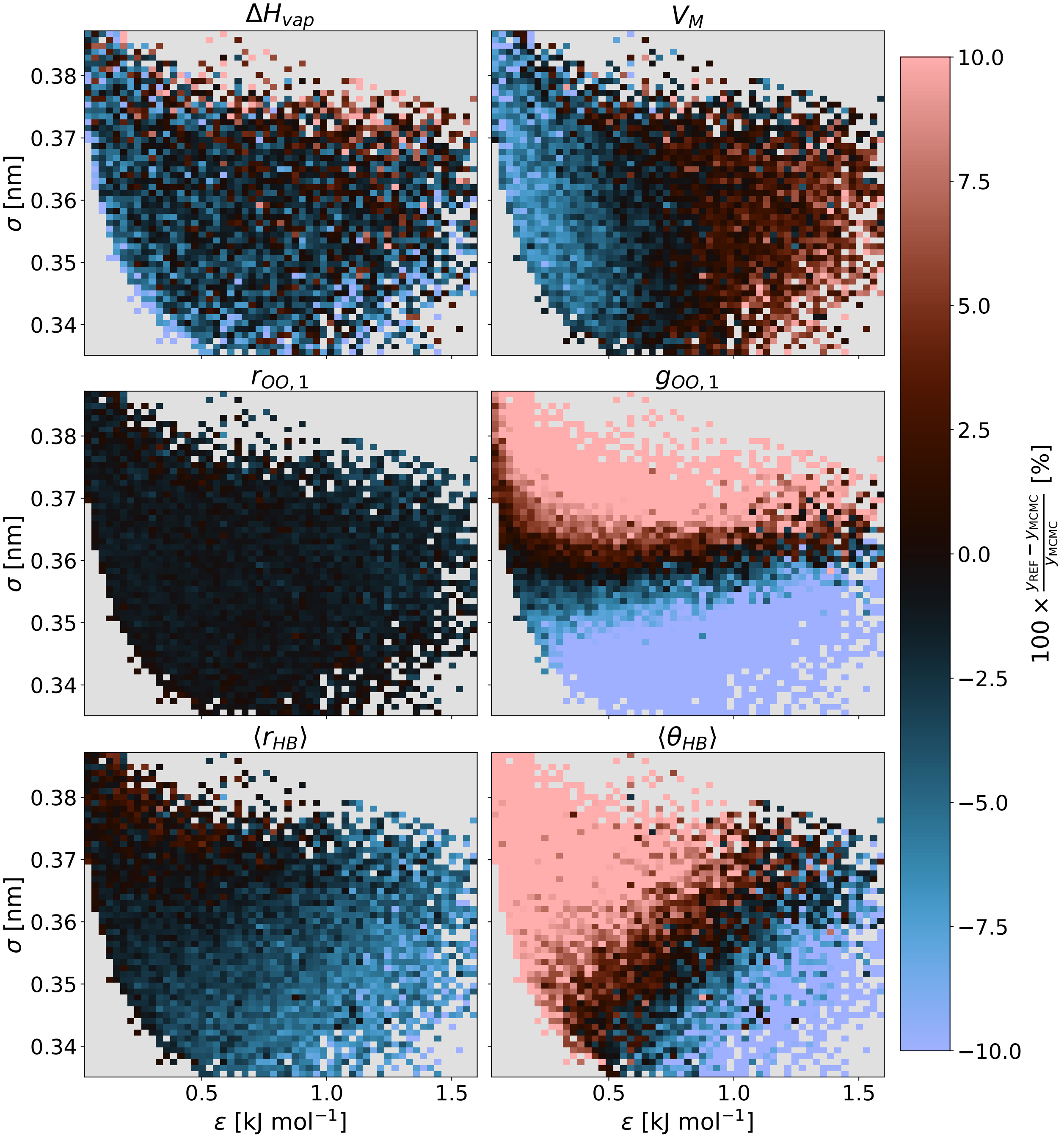

Figure S1: Median relative deviations from experiment for the six observables used in the Wang-Buckingham inference, shown across the sampled (ϵ, σ) parameter space. Each panel reports the percent deviation from the experimental reference for one observable. The distinct low-deviation bands reveal that different observables are best reproduced in different regions of parameter space, highlighting the competing constraints they impose on the model.

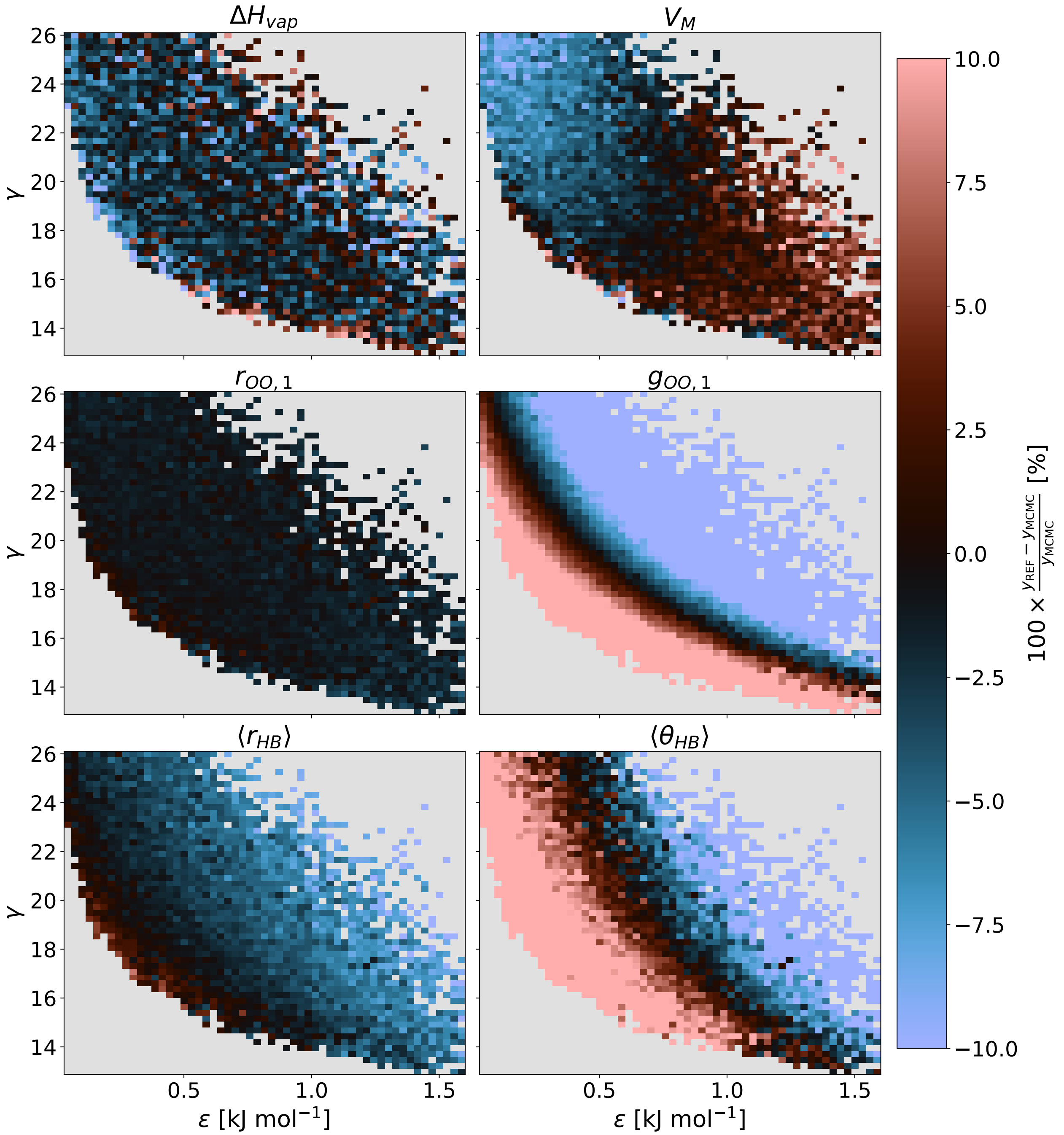

Figure S2: Median relative deviations from experiment for the six observables used in the Wang-Buckingham inference, shown across the sampled (ϵ, γ) parameter space. Each panel reports the percent deviation from the experimental reference for one observable. The distinct low-deviation bands reveal that different observables are best reproduced in different regions of parameter space, highlighting the competing constraints they impose on the model.

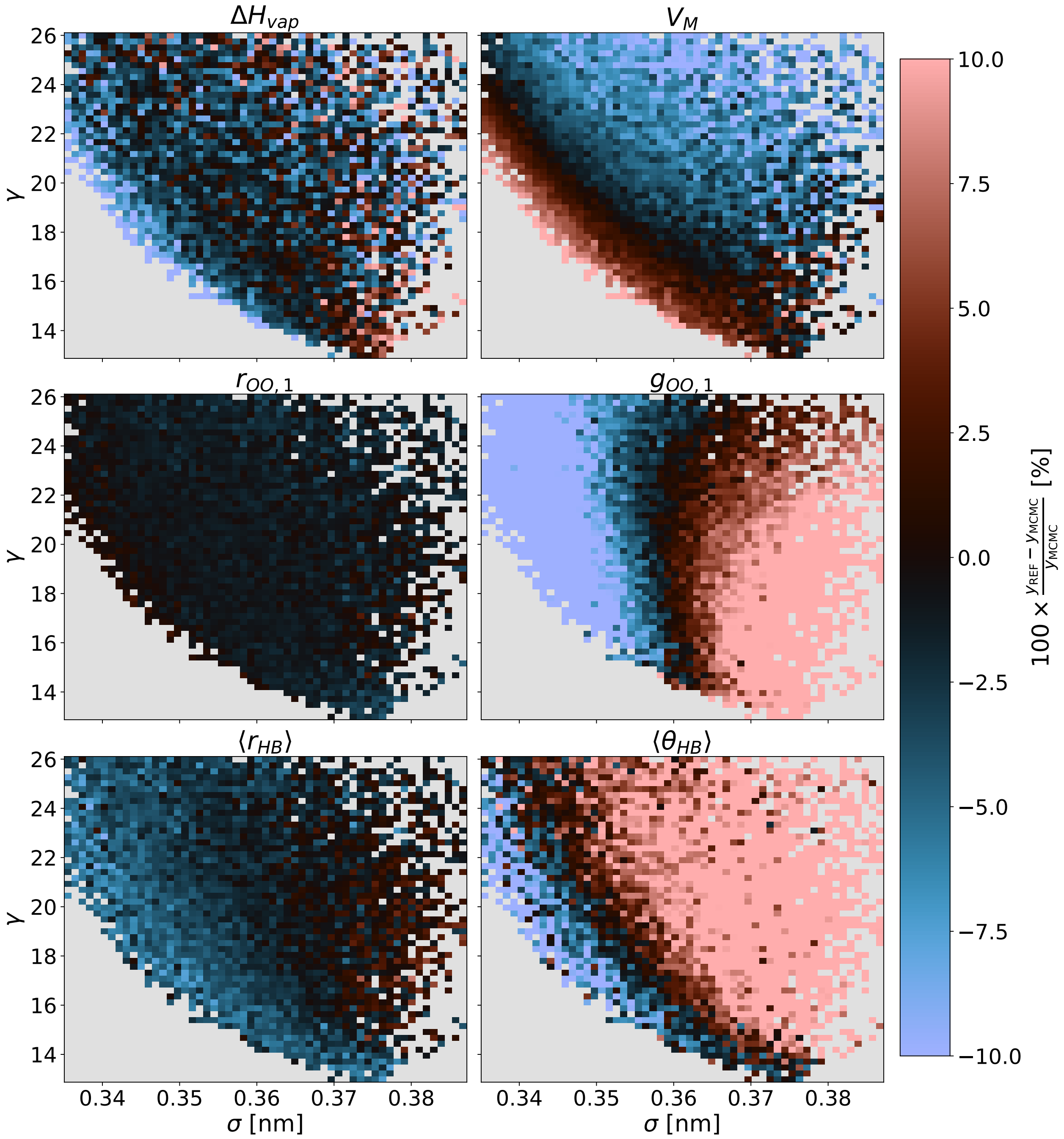

Figure S3: Median relative deviations from experiment for the six observables used in the Wang-Buckingham inference, shown across the sampled (σ, γ) parameter space. Each panel reports the percent deviation from the experimental reference for one observable. The distinct low-deviation bands reveal that different observables are best reproduced in different regions of parameter space, highlighting the competing constraints they impose on the model.

References

(1) Yeh, I.-C.; Hummer, G. System-size dependence of diffusion coefficients and viscosities from molecular dynamics simulations with periodic boundary conditions. *J. Phys. Chem. B.* **2004**, *108*, 15873–15879.

(2) Teng, X.; Yu, W.; MacKerell Jr, A. D. Revised 4-Point water model for the classical Drude oscillator polarizable force field: SWM4-HLJ. *J. Chem. Theory Comput.* **2024**, *20*, 10034–10044.

(3) Waheed, Q.; Edholm, O. Quantum Corrections to Classical Molecular Dynamics Simulations of Water and Ice. *J. Chem. Theory Comput.* **2011**, *7*, 2903–2909.
